# Vitamin D supplementation and the outcomes of critically ill adult patients: a systematic review and meta-analysis of randomized controlled trials

**DOI:** 10.1038/s41598-020-71271-9

**Published:** 2020-08-31

**Authors:** Shao-Huan Lan, Chih-Cheng Lai, Shen-Peng Chang, Li-Chin Lu, Shun-Hsing Hung, Wei-Ting Lin

**Affiliations:** 1 School of Pharmaceutical Sciences and Medical Technology, Putian University, Putian, 351100 China; 2 Department of Internal Medicine, Kaohsiung Veterans General Hospital, Tainan Branch, Tainan, Taiwan; 3 Yijia Pharmacy, Tainan, 70846 Taiwan; 4 School of Management, Putian University, Putian, 351100 China; 5 Division of Urology, Department of Surgery, Chi-Mei Hospital, Chia Li, Tainan, Taiwan; 6 Department of Orthopedic, Chi Mei Medical Center, Tainan, 71004 Taiwan

**Keywords:** Diseases, Nutrition disorders

## Abstract

This meta-analysis assessed the association between vitamin D supplementation and the outcomes of critically ill adult patients. A literature search was conducted using the PubMed, Web of Science, EBSCO, Cochrane Library, Ovid MEDLINE, and Embase databases until March 21, 2020. We only included randomized controlled trials (RCTs) comparing the efficacy of vitamin D supplementation with placebo in critically ill adult patients. The primary outcome was their 28-day mortality. Overall, 9 RCTs with 1867 patients were included. In the pooled analysis of the 9 RCTs, no significant difference was observed in 28-day mortality between the vitamin D supplementation and placebo groups (20.4% vs 21.7%, OR, 0.73; 95% CI, 0.46–1.15; *I*^2^ = 51%). This result did not change as per the method of vitamin D supplementation (enteral route only: 19.9% vs 18.2%, OR, 1.19; 95% CI, 0.88–1.57; *I*^2^ = 10%; intramuscular or intravenous injection route: 25.6% vs 40.8%, OR, 0.48; 95% CI, 0.21–1.06; *I*^2^ = 19%) or daily dose (high dose: 20.9% vs 19.8%, OR, 0.83; 95% CI, 0.51–1.36; *I*^2^ = 53%; low dose: 15.6% vs 21.3%, OR, 0.74; 95% CI, 0.32–1.68; *I*^2^ = 0%). No significant difference was observed between the vitamin D supplementation and placebo groups regarding the length of ICU stay (standard mean difference [SMD], − 0.30; 95% CI, − 0.61 to 0.01; *I*^2^ = 60%), length of hospital stay (SMD, − 0.17; 95% CI, − 041 to 0.08; *I*^2^ = 65%), and duration of mechanical ventilation (SMD, − 0.41; 95% CI, − 081 to 0.00; *I*^2^ = 72%). In conclusion, this meta-analysis suggested that the administration of vitamin D did not provide additional advantages over placebo for critically ill patients. However, additional studies are needed to confirm our findings.

## Introduction

Vitamin D, a fat-soluble vitamin, is an essential nutrient in bone metabolism and calcium and phosphorus homeostasis. However, the system of vitamin D is complex, in which some novel pathways have been found for host response to vitamin D treatment including non-canonical pathways of vitamin D activation^1,2^ leading to production of non- or low-calcemic analogs^3^ and of lumisterol activation^4^. In clinical practice, vitamin D is used for the treatment of hyperproliferative skin diseases, hyperparathyroidism, and osteoporosis. Vitamin D also exhibits other non-skeletal pleiotropic properties, such as immunomodulatory, antimicrobial, cardiovascular, and muscular effects. Therefore, vitamin D deficiency is associated with many diseases including tuberculosis, nonalcoholic fatty liver disease, cardiovascular disease, and metabolic syndrome^5–7^. In the United States, adults aged 20–39 years are at the highest risk of vitamin D deficiency (the prevalence: 7.6%; 95% CI: 6.0–9.6%)^8^. One study conducted in Europe showed that 13.0% of 55,844 European individuals showed average serum 25(OH)D concentrations of < 30 nmol/L^9^. In China, 30.6% of elderly people have vitamin D deficiency^10^.

In addition to its prevalence in the general population, vitamin D deficiency is common among critically ill patients. Lee et al. showed that 64.5% (n = 120) of critically ill surgical patients had serum 25(OH)D concentrations of < 20 nmol/L^11^, and Higgins et al. reported that 26% (50/196) of patients admitted to a medical/surgical intensive care unit (ICU) had vitamin D levels of ≤ 30 nmol/L^12^. A retrospective cohort study showed that 54% (65/121) of patients with severe sepsis or septic shock had vitamin D levels lower than 15 mg/mL^13^, and another prospective multicenter study demonstrated vitamin D deficiency in 78.8% (197/250) of patients^14^. Furthermore, several studies document that vitamin D deficiency could be associated with poor outcomes in critically ill patients^12,13,15–18^. To improve the outcomes of critically ill patients, vitamin D supplementation was proposed for ICU patients. Several randomized controlled trials (RCTs) were conducted to investigate the effects of vitamin D supplementation on the outcomes of critically ill patients. However, their results are conflicting^19–28^. Some studies showed that vitamin D supplementation demonstrated positive effects by decreasing the length of hospital stay^23^, duration of mechanical ventilation (MV)^26,27^, and mortality rate^24,27^. However, some studies^11,20,21,25,29,30^ reported no change in the outcomes of critically ill patients. Even 2 meta-analyses, the included studies of which were published before 2017^31,32^, provided inconsistent findings. Since 2017, four more RCTs^24–27^ have reported their findings. Therefore, we conducted an updated meta-analysis of RCTs to assess the association between vitamin D supplementation and the outcomes of critically ill patients.

## Methods

### Study search and selection

We conducted a literature review using the databases of PubMed, Embase, Web of Science, EBSCO, Cochrane Library, Ovid Medline, Embase, and Proquest until March 21, 2020. The following search terms were used: “intensive care” “ICU,” “critically-ill,” “vitamin D,” “calcitriol,” “Cholecalciferol*,” “ergocalciferol*,” and “RCT.” Our meta-analysis only included RCTs that investigated the clinical efficacy of vitamin D supplementation compared with placebo for critically ill adult patients. The supplementation could be done in different ways, such as oral, enteral, or parenteral vitamin D administration as 1, 25-dihydroxyvitamin D (calcitriol) or 25-hydroxyvitamin D (cholecalciferol). Two authors (Lan SH and Chang SP) searched for related studies and examined the risk of bias in each study using the Cochrane Risk of Bias Assessment tool^33^. When they had different opinions, a third author (Lai CC) helped resolve the issue. Data, including the year of publication, study design, study location and duration, demographic characteristics of critically ill patients, regimen of vitamin D, patient outcomes, and adverse events, were extracted from each included study. This study followed the Preferred Reporting Items for Systematic Reviews and Meta-analyses (PRISMA) reporting guidelines.

### Definitions and outcomes

Critically ill patients were defined as that the patients with acute respiratory failure required mechanical ventilation or the patients required ICU hospitalization. The primary outcome of the current study was the patients’ 28-day mortality. If data on 28-day mortality were not available, hospital mortality was used in the meta-analysis. Secondary outcomes included the length of ICU and hospital stay and the duration of MV. Doses of ≥ 300,000 and < 300,000 IU of vitamin D daily were defined as high and low doses, respectively, as per a previous study^34^.

### Statistical analysis

We used Review Manager software (The Cochrane Collaboration 2008, Copenhagen) to develop a random-effects model and derive the pooled estimates and their associated 95% CIs. The odds ratio (OR) was used to evaluate the outcome of 28-day mortality. Standardized mean differences (SMDs) and 95% CIs were computed for continuous variables including length of ICU and hospital stay and the duration of MV.

## Results

### Study selection

**Figure 1:**
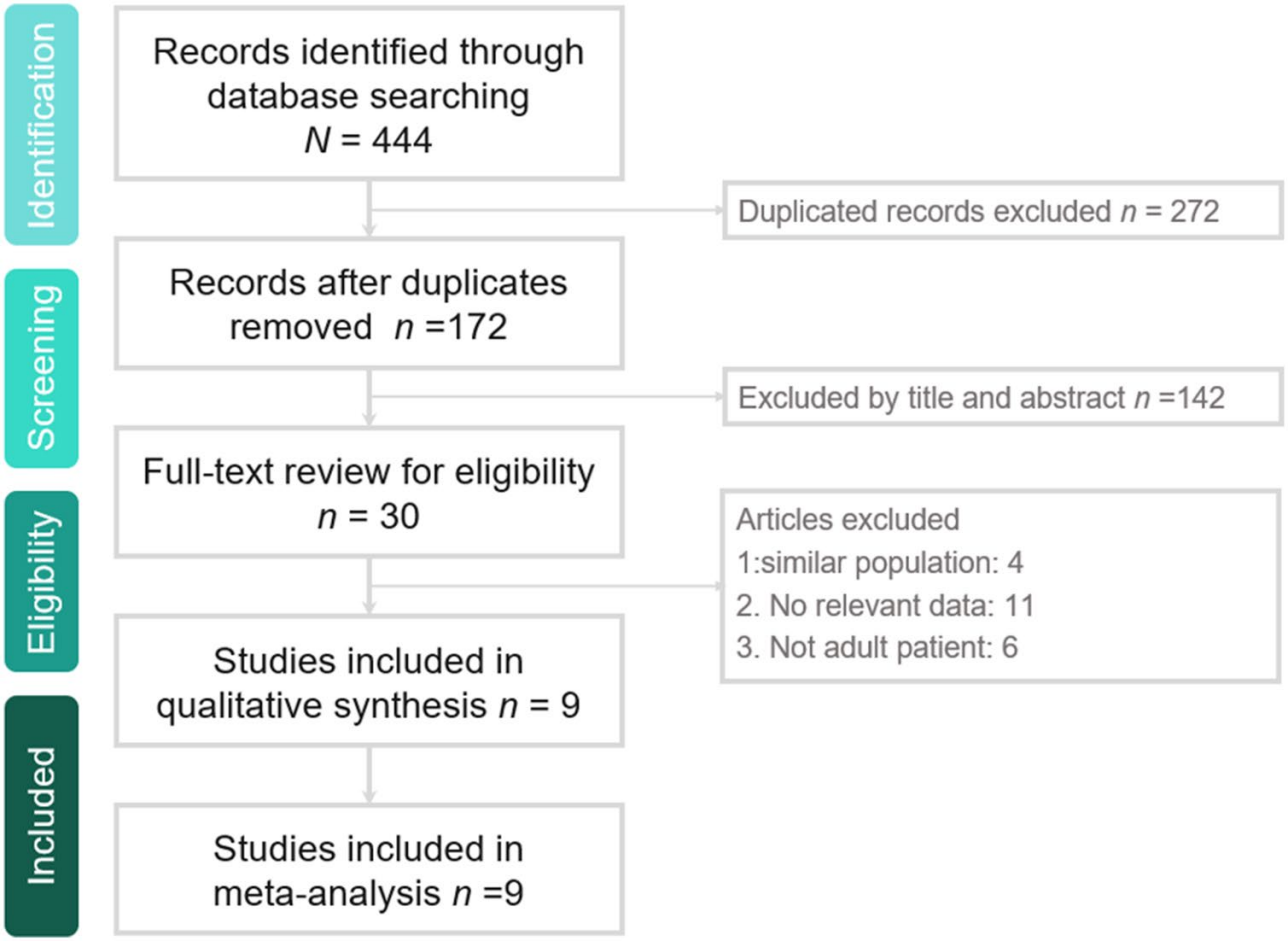
Flowchart of study selection.

Our search yielded 444 studies in total from online databases from PubMed (n = 56), Web of Science (n = 71), EBSCO (n = 27), Cochrane library (n = 107), Ovid MEDLINE (n = 74), and Embase (n = 85); 272 duplicate studies were excluded. The remaining 172 articles were identified. Moreover, 142 studies were found to be irrelevant after the title and abstract were screened, and 19 studies were found to be irrelevant after the full text was screened. Eventually, 9 RCTs^19–27^ were included in this meta-analysis (Fig. 1, Appendix 1).

### Study characteristics

**Figure 2:**
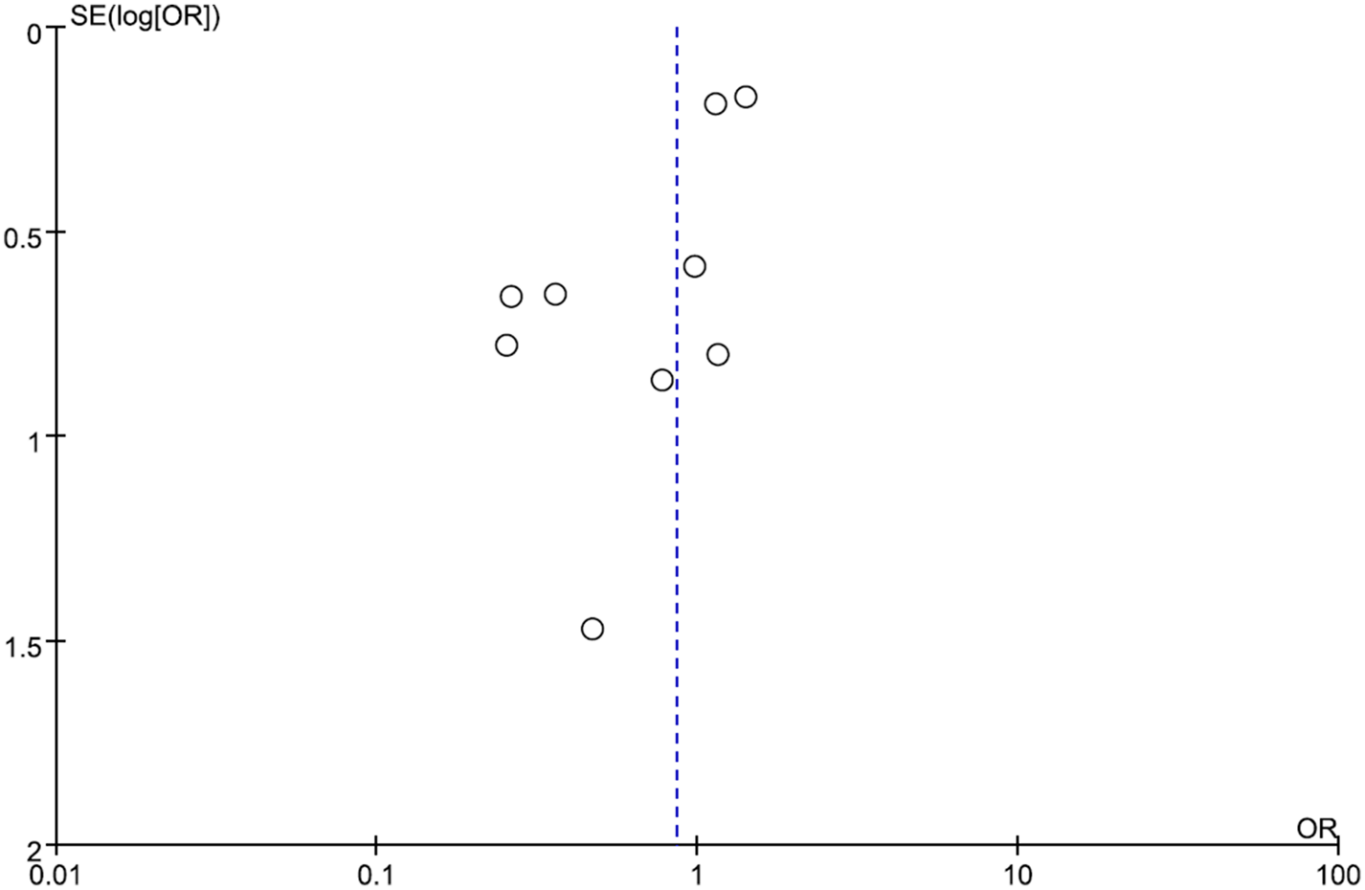
Funnel plot for comparison.

Five RCTs^19,21,22,26,27^ were conducted in a single center, and three RCTs^20,23,24^ were conducted in two centers. Only one RCT^25^ was a multicenter study (Table 1). Four studies^20,22,23,25^ were conducted in the United States, three^24,26,27^ were in Iran, and two^19,21^ in Austria. Overall, these nine RCTs included a total of 1,640 critically ill patients. Three RCTs^19,21,25^ only enrolled patients with vitamin D levels ≤ 20 ng/mL, and two studies^26,27^ enrolled patients with vitamin D levels ≤ 20 ng/mL. Vitamin D was administered through the enteral route in five studies^19,21–23,25^, through intravenous or intramuscular injection in three^20,24,27^, and both routes in one^26^. Single-dose regimens of vitamin D were used in six studies^19,20,22,24,25,27^ and multidose regimens in three studies^21,23,26^. Almost all risks of bias were low in each study. The study by Miroliaee et al.^24^ and Hasanloei et al.^26^ had a high risk of allocation, and detection bias. The publication bias is shown in a funnel plot (Fig. 2).

**Table 1:** Characteristics of the randomized placebo-controlled trials included in the meta-analysis.

Study, publish year	Study sites	Study duration	No of patients	Study population	Intervention
Amrein et al., 2011	Single center in Austria	2009–2010	25	Adult patients expected to stay in the ICU for 48 h or more, and had a 25-hydroxyvitamin D level ≤ 20 ng/mL	Single enteral dose of vitamin D3 540,000 IUs
Leaf et al., 2014	Two centers in USA	2013	67	ICU adult patients with severe sepsis or septic shock and presence of an arterial or central venous catheter	Single intravenous dose of calcitriol 2 μg
Amrein et al., 2014	Single center in Austria	2012–2015	475	Patients who were 18 years or older expected to stay in the ICU for 48 h or more, and had a 25-hydroxyvitamin D level ≤ 20 ng/mL	Single enteral dose of vitamin D3 540,000 IUs followed by monthly maintenance doses of 90,000 IU for 5 months
Quraishi et al., 2015	Single center in USA	2014	30	Adult patients admitted to medical or surgical ICU and with 24 h of new onset sepsis	Single enteral dose of vitamin D3 200,000 IU or 400,000 IUs
Han et al., 2016	Two centers in USA	NR	30	Adult patients received care in ICU, expected to require MV for ≥ 72 h and expected to survive and remain in ICU for ≥ 96 h	Different vitamin D3 enteral doses divided more than 5 consecutive days (50,000 IU or 100,000 IU daily)
Miroliaee et al., 2017	Two centers in Iran	2014–2015	46	Adult patients who had been diagnosed wtih ventilator-associated pneumonia and 25-hydroxyvitamin D level ≤ 30 ng/mL	300,000 IUs of intramuscular vitamin D
Ginde et al., 2019	44 centers in USA	2017–2018	1,078	Adult patients admitted to ICU and had 25-hydroxyvitamin D level ≤ 20 ng/mL	a single enteral dose of 540,000 IU1
Hasanloei et al., 2019	Single center in Iran	2017–2018	72	Traumatic injury admitted to ICU with a 25(OH)D serum level between 10 and 30 ng/mL	Oral 50,000 IU cholecalciferol daily for 6 days, or one intramuscular injection of 300,000 IU of cholecalciferol
Miri et al., 2019	Single center in Iran	NA	40	Mechanically ventilated patient admitted to ICU	intramuscular injection of 300,000 IU vitamin D

*NA* not applicable.

### Primary outcome

**Figure 3:**
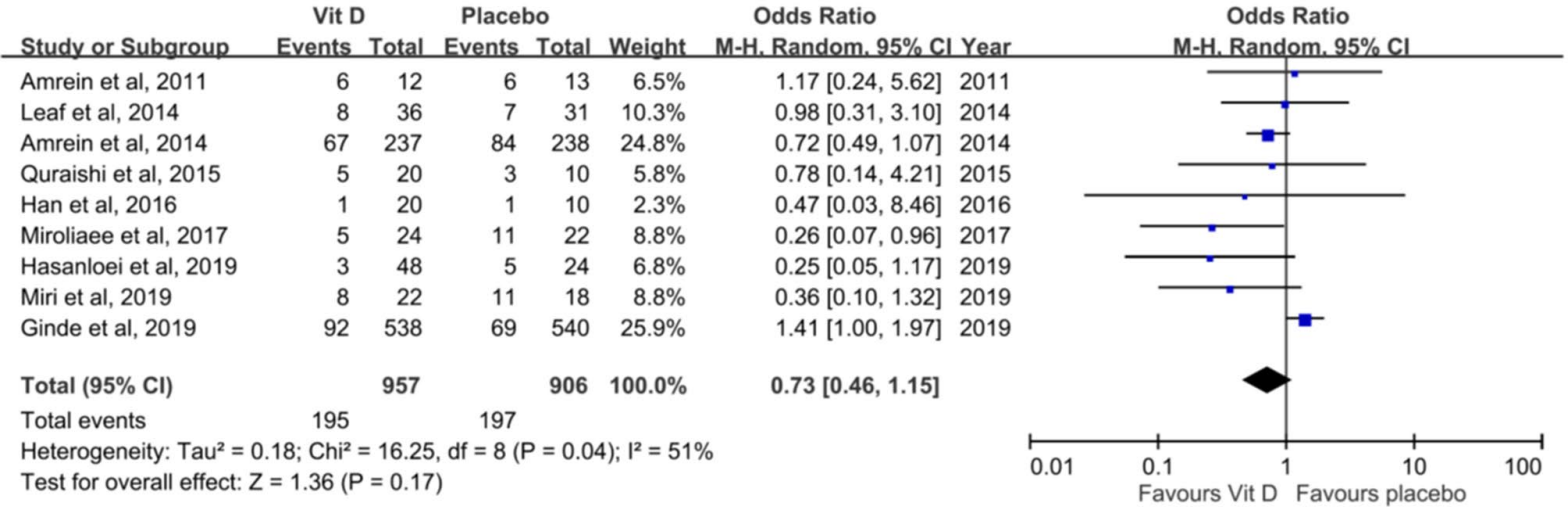
Effect of vitamin D on 28-day mortality.

In the pooled analysis of nine RCTs, no significant difference was observed in 28-day mortality between the vitamin D supplementation and placebo groups (20.4% vs 21.7%, OR, 0.73; 95% CI, 0.46–1.15; *I*^2^ = 51%, Fig. 3). A sensitivity analysis performed after excluding individual studies did not change this result. Similarly, in the subgroup analysis of RCTs that enrolled only patients with vitamin D deficiency, no significant differences were observed in mortality (21.4% vs 19.7%, OR, 0.93; 95% CI, 0.57–1.52; *I*^2^ = 58%). This result did not change as per the method of vitamin D supplementation (enteral route only: 19.9% vs 18.2%, OR, 1.19; 95% CI, 0.88–1.57; *I*^2^ = 10%; intramuscular or intravenous injection route: 25.6% vs 40.8%, OR, 0.48; 95% CI, 0.21–1.06; *I*^2^ = 19%) or the daily dose (high dose: 20.9% vs 19.8%, OR, 0.83; 95% CI, 0.51–1.36; *I*^2^ = 53%; low dose: 15.6% vs 21.3%, OR, 0.74; 95% CI, 0.32–1.68; *I*^2^ = 0%). The similar trend was observed for subgroup with baseline vitamin D deficiency (19.3% vs 19.1%, OR, 0.80; 95% CI, 0.48–1.46; *I*^*2*^ = 63%). Finally, although the studies conducted from 2016 to 2019 seems to have a better outcome than those from 2011 to 2015, the pooled analysis of 5 studies conducted from 2016 to 2019 still did not show the significant difference between vit D and placebo group (OR, 0.50; 95% CI, 0.18–1.34, I2 = 0.70%).

### Secondary outcome

**Figure 4:**
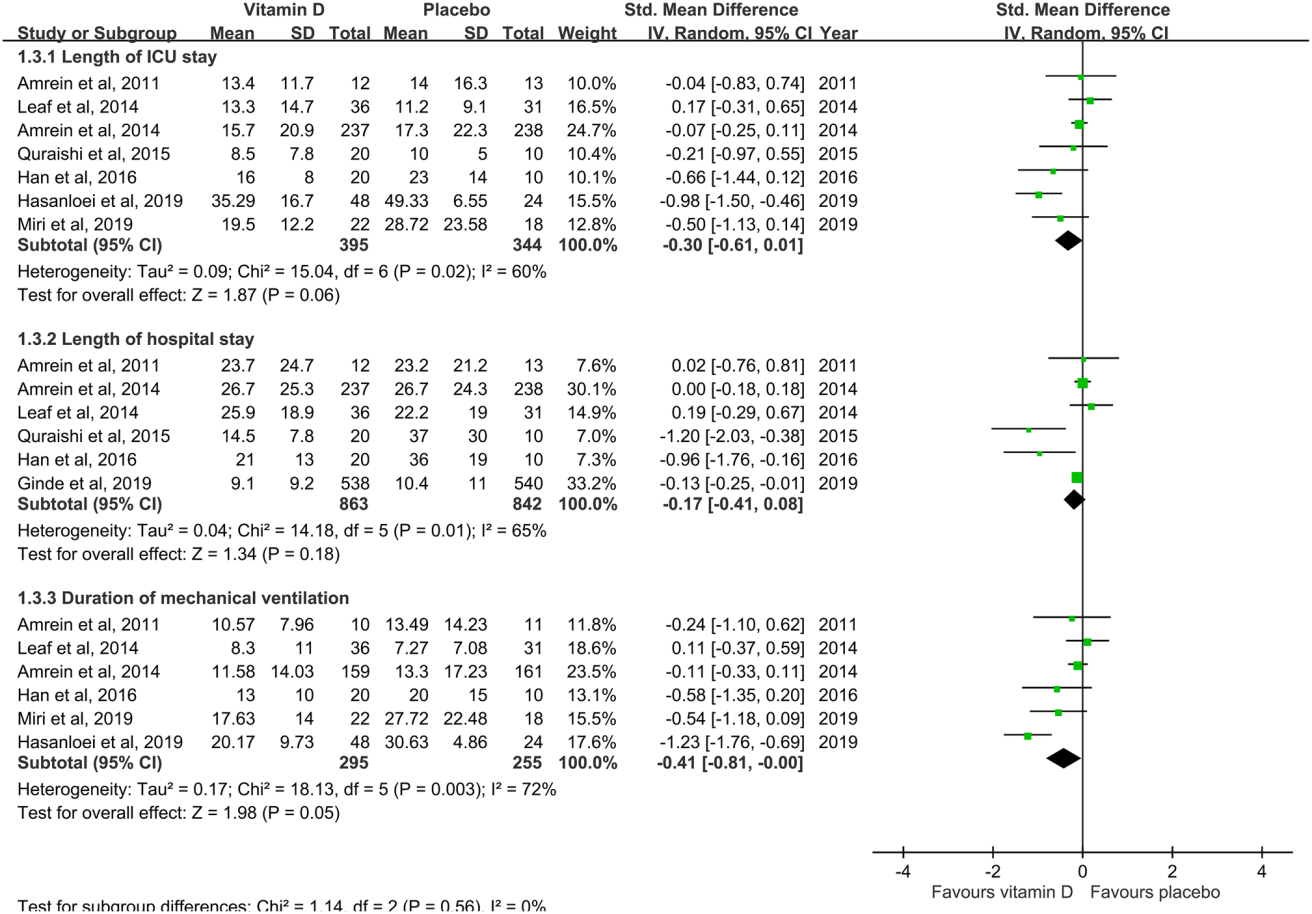
Effect of vitamin D on length of intensive care unit and hospital stay and the duration of mechanical ventilation.

The pooled analysis of seven studies^19–23,26,27^ reported no significant difference in the length of ICU stay between the vitamin D supplementation and placebo groups (SMD, − 0.30; 95% CI, − 0.61 to 0.01; *I*^2^ = 60%)(Fig. 4). Analysis of six studies^19–23,25^ reported no significant difference in the length of hospital stay between the vitamin D supplementation and placebo groups (SMD, − 0.17; 95% CI, − 041 to 0.08; *I*^2^ = 65%)(Fig. 4). Six studies^19–21,23,26,27^ reported no significant difference in the duration of MV between both groups (SMD, − 0.41; 95% CI, − 0.81 to 0; *I*^2^ = 72%)(Fig. 4). A subgroup analysis showed that high-dose vitamin D supplementation did not change length of ICU stay (SMD, − 1.82; 95% CI, − 5 to 1.35; *I*^2^ = 99%), length of hospital stay (SMD, − 0.09; 95% CI, − 0.25 to 0.06; *I*^2^ = 31%), and duration of MV (SMD, − 0.42; 95% CI, − 0.92 to 0.07; *I*^2^ = 70%). Similarly, low-dose vitamin D did not change length of ICU stay (SMD, 0.29; 95% CI, − 2.43 to 3; *I*^2^ = 97%), length of hospital stay (SMD, − 0.54; 95% CI, − 1.45 to 0.36; *I*^2^ = 78%), and duration of MV (SMD, − 0.65; 95% CI, − 1.66 to 0.37; *I*^2^ = 87%).

## Discussion

This meta-analysis included nine RCTs with 1867 patients to compare the efficacy and safety of vitamin D supplementation with placebo in critically ill patients. The outcome was numerically better in the vitamin D supplementation group than control group, which may suggest biologically significant trends favoring vitamin D supplementation, however, these differences did not reach statistical significance. Overall, our results suggested that vitamin D supplementation did not significantly improve the outcomes of critically ill patients, which was supported by the following evidence. First, 28-day mortality did not change with vitamin D supplementation in the pooled analysis of 9 RCTs. Second, this difference remained unchanged in the sensitivity test. Third, we also found no significant improvement in the mortality of critically ill patients with vitamin D deficiency in the subgroup analysis. Fourth, compared with the placebo group, we found no significant difference in mortality in the vitamin D supplementation group with either enteral or injection administration of vitamin D and with administration of low- or high-dose vitamin D. Finally, we assessed the effect of vitamin D on the length of ICU and hospital stay and MV duration and found no significant difference between the vitamin D supplementation and placebo groups. Moreover, no difference was observed in the subgroup analysis of high and low doses of vitamin D. The aforementioned findings indicate that compared with placebo, the vitamin D supplementation is not associated with lower mortality in critically ill patients.

Our findings are consistent with those of a meta-analysis by Langlois et al.^32^, in which they included six RCTs of 695 patients, and they found that vitamin D did not reduce the mortality, length of ICU and hospital stay, and period on a ventilator. However, another meta-analysis by Putzu et al.^31^ including 7 studies of 716 patients between 2011 and 2016 showed that vitamin D supplementation was associated with lower mortality compared with placebo (OR, 0.70; 95% CI, 0.50–0.98, *I*^2^ = 0). This difference could be because we included a recent large-scale study of more than 1,000 patients by Ginde et al.^25^, in which the administration of high-dose vitamin D did not provide an additional benefit with respect to clinical outcomes, including mortality. Moreover, the data of clinical outcomes in the analysis by Putzu et al.^31^ had been reported by only 3 of 4 studies, which may limit the generalizability of their findings. Conversely, our study included more patients, more updated studies, and more subgroup analyses than previous studies^31,32^. In addition, all of our analyses showed consistent findings. Therefore, our findings provide stronger evidence regarding the effect of vitamin D supplementation on the outcomes of critically ill patients than previous studies.

However, this study had several limitations. Although this study focused on critically ill patients, their clinical characteristics are heterogeneous. Some were admitted to the ICU for traumatic injury, and some had ventilator-associated pneumonia. The criteria of vitamin D deficiency varied across studies, and the disease severity of the study patients also differed. Therefore, potential positive effects of vitamin D supplementation on the patient outcomes could not be found in this pooled analysis. In addition, only limited studies reported the vit D3 level after treatment and their level increased after treatment. Thus, we cannot assess the association between the level of vitamin D after treatment and the clinical outcome. Further studies are warranted to discover specific populations who can benefit from vitamin D supplementation^35^.

## Conclusion

This meta-analysis suggested that the administration of vitamin D did not provide additional advantages over placebo for critically ill patients. However, additional studies are needed to confirm our findings.

## Supplementary information

Supplementary information.
