# Supplementary material for: Vitamin D supplementation and the outcomes of critically ill adult patients: a systematic review and meta-analysis of randomized controlled trials
Source: Sci Rep. 2020 Aug 31;10:14261. doi: 10.1038/s41598-020-71271-9 (PMC7459294; doi:10.1038/s41598-020-71271-9)
Supplement: Supplementary file 1 — Supplementary information. [file 41598_2020_71271_MOESM1_ESM.docx]

**Vitamin D Supplementation and the Outcomes of Critically Ill Adult Patients: A Systematic Review and Meta-Analysis of Randomized Controlled Trials**

Shao-Huan Lan, PhD,^1,#^ Chih-Cheng Lai, MD,^2,#^ Shen-Peng Chang, PhD,^3^ Li-Chin Lu, PhD,^4^ Shun-Hsing Hung, MD,^5,*^ Wei-Ting Lin, MD^6, *^

^1^ School of Pharmaceutical Sciences and Medical Technology, Putian University, Putian 351100, China.

^2^Department of Internal Medicine, Kaohsiung Veterans General Hospital, Tainan Branch, Tainan, Taiwan,

^3^Yijia Pharmacy, Tainan 70846, Taiwan.

^4^ School of Management, Putian University, Putian 351100, China.

^5^Division of Urology, Department of Surgery, Chi-Mei Hospital, Chia Li, Tainan, Taiwan,

^6^Department of Orthopedic, Chi Mei Medical Center, Tainan 71004, Taiwan.

**Appendix 1. Search strategy**

| **PubMed search strategy – last searched on March 21, 2020** | | **Results** |
| --- | --- | --- |
| **1** | **Search (intensive care[Title/Abstract]) OR ICU[Title/Abstract] OR critically-ill[Title/Abstract]** | **181047** |
| **2** | **Search (((Vitamin-D[Title/Abstract]) OR calcitriol[Title/Abstract]) OR cholecalciferol*[Title/Abstract]) OR ergocalciferol*[Title/Abstract]** | **64833** |
| **3** | **Search (RCT[Title/Abstract]) OR random*[Title/Abstract]** | **1108073** |
| **4** | **Search (((((RCT[Title/Abstract]) OR random*[Title/Abstract]))) AND (((((Vitamin-D[Title/Abstract]) OR calcitriol[Title/Abstract]) OR Cholecalciferol*[Title/Abstract]) OR Ergocalciferol*[Title/Abstract]))) AND (((intensive care[Title/Abstract]) OR ICU[Title/Abstract] OR critically-ill[Title/Abstract]))** | **56** |

| **Web of Science search strategy – last searched on March 21, 2020** | | **Results** |
| --- | --- | --- |
| **1** | **TS= (Vitamin-D) OR TS= (Calcitriol) OR TS= (Cholecalciferol*) OR TS= (Ergocalciferol*)** | **83997** |
| **2** | **TS= (Intensive-care) OR TS= (ICU) OR TS= (critical-ill)** | **155430** |
| **3** | **TS= (RCT) OR TS= (Random*)** | **1528026** |
| **4** | **#3 AND #2 AND #1** | **71** |

| **EBSCO search strategy – last searched on March 21, 2020** | | **Results** |
| --- | --- | --- |
| **1** | **AB Vitamin-D OR AB Calcitriol OR AB Cholecalciferol* OR AB Ergocalciferol*** | **24531** |
| **2** | **AB Intensive-care OR AB ICU OR AB critical-ill** | **64192** |
| **3** | **AB RCT OR AB Random*** | **497166** |
| **4** | **S1 AND S2 AND S3** | **27** |

| **Cochrane Library search strategy – last searched on March 21, 2020** | | **Results** |
| --- | --- | --- |
| **1** | **(Vitamin-D):ti,ab,kw OR (Calcitriol):ti,ab,kw OR (Cholecalciferol*):ti,ab,kw OR (Ergocalciferol*):ti,ab,kw** | **12718** |
| **2** | **(Intensive-care):ti,ab,kw OR (ICU):ti,ab,kw OR (critical-ill):ti,ab,kw** | **30113** |
| **3** | (RCT):ti,ab,kw OR (random*):ti,ab,kw | **980586** |
| **4** | #1 AND #2 AND #3 | **107** |

| **Ovid Medline search strategy – last searched on March 21, 2020** | | **Results** |
| --- | --- | --- |
| **1** | **(Vitamin-D or Calcitriol or Cholecalciferol* or Ergocalciferol*).ab.** | **69795** |
| **2** | **(intensive-care or ICU or critical-ill).ab.** | **213943** |
| **3** | **(RCT or Random*).ab.** | **1492164** |
| **4** | **1 and 2 and 3** | **74** |

| **Embase search strategy – last searched on March 21, 2020** | | **Results** |
| --- | --- | --- |
| **1** | **'vitamin d':ti,ab,kw OR calcitriol:ti,ab,kw OR cholecalciferol*:ti,ab,kw OR ergocalciferol*:ti,ab,kw** | **100431** |
| **2** | **icu:ti,ab,kw OR 'intensive care':ti,ab,kw OR 'critical ill':ti,ab,kw** | **260872** |
| **3** | **rct:ti,ab,kw OR random*:ti,ab,kw** | **1506265** |
| **4** | **#1 AND #2 AND #3** | **85** |
